# Is the "Glasgow effect" of cigarette smoking explained by socio-economic status?: A multilevel analysis

**DOI:** 10.1186/1471-2458-9-245

**Published:** 2009-07-17

**Authors:** Linsay Gray, Alastair H Leyland

**Affiliations:** 1 Social and Public Health Sciences Unit, Medical Research Council, Glasgow, UK

## Abstract

**Background:**

The Glasgow area has elevated levels of deprivation and is known for its poor health and associated negative health-related behaviours, which are socially patterned. Of interest is whether high smoking rates are explained by the area's socio-economic profile.

**Methods:**

Data on age, sex, current/previous smoking status, area deprivation, social class, education, economic activity, postcode sector, and health board region were available from Scottish Health Surveys conducted in 1995, 1998 and 2003. Multilevel logistic regression models were applied by sex, unadjusted and adjusted for age, survey year, and socio-economic factors, accounting for geographical hierarchy and missing data.

**Results:**

Compared with the rest of Scotland, men living in Greater Glasgow were 30% and women 43% more likely to smoke [odds ratio (OR) = 1.30, (95% CI = 1.08–1.56) and (OR = 1.43, CI = 1.22–1.68), respectively] before adjustment. In adjusted results, the association between living in Greater Glasgow and current smoking was attenuated [OR = 0.92, CI = 0.78–1.09 for men, and OR = 1.08, CI = 0.94–1.23 for women; results based on multiply imputed data to account for missing values remained borderline significant for women]. Accounting for individuals who had been told to give up smoking by a medical person/excluding ex-smokers did not alter results.

**Conclusion:**

High levels of smoking in Greater Glasgow were attributable to its poorer socio-economic position and the strong social patterning of smoking. Tackling Glasgow's, and indeed Scotland's, poor health must involve policies to alleviate problems associated with poverty.

## Background

Smoking is the major preventable cause of death and disability in the developed world. It is known to impact negatively on the risk of heart disease, stroke and cancer, especially of the lung.[1] Lung cancer rates in Scotland were amongst the highest in the world in the early 1990s [2] and the Glasgow area has one of the highest incidences of cardiovascular disease in the world.[3] Although declining, [4] prevalence of cigarette smoking in Scotland is higher than in other parts of the UK, [4,5] and the problem is particularly severe in the Glasgow area.[6] High rates have been implicated in the city's poor health record, [7] with an estimated one in five deaths being attributable to the habit. Smoking is socially patterned with far higher prevalence in low socio-economic status groups [8] and is the leading cause of health inequalities.[7,9] The socio-economic composition of Glasgow differs to that of the rest of Scotland and the UK with overall higher levels of deprivation; [10] in some of the most deprived parts of Glasgow almost two-thirds of the population currently smoke [10] As well as varying by area deprivation and social class, smoking is strongly positively correlated with poor education attainment [11] and unemployment.[12,13]

Patterns of ill-health that characterise the Glasgow area may be due to some unknown 'Glasgow effect' adversely affecting the city's health beyond that explained by conventional risk factors. The extent to which elevated levels of smoking in Glasgow can be accounted for by socio-economic differences, or are truly attributable to a separate 'Glasgow effect', has not been addressed.

Since smoking patterns vary with age and sex as well as over time, [4] investigation should take account of such demographics. Associations of health and related behaviours with geographical area can be different for men and women, [14] thus it is important to stratify by sex. Smoking has been shown to cluster geographically in Scotland [15,16] and even after controlling for social class and income as well as gender and age, prevalence is independently associated with neighbourhood of residence.[17] In any regional analysis of smoking, context in terms of smaller geographical area should be taken into account.

Since individuals with smoking-related health problems may have stopped smoking on medical advice, [18] and there are geographical variations in the prevalence of such health problems, [19] any geographical analysis should be assessed for robustness to the impact of ex-smokers quitting the habit on medical advice.

The aim of this paper is to investigate whether differences in smoking status between adults living in the Glasgow area and the rest of Scotland can be explained by socio-economic factors, taking into account the additional effects of context and prior smoking status – considered both in terms of receipt of medical advice to stop smoking and by excluding those who stopped following medical advice.

## Methods

Data are from three cross-sectional Scottish Health Surveys in which nationally representative samples of the population living in private households in Scotland were interviewed in person in 1995, [20] 1998 [21] and 2003.[22] In each, around one third (312) of the postcode sectors of Scotland was sampled from all 15 health boards in Scotland, including Greater Glasgow, as was the case at the times of surveys. Postcode sectors are small areas with total population averaging 5,000, although there is considerable variety in size. Altogether, data on 25,127 individuals were available from the 1995 (7,932), 1998 (9,047) and 2003 (8,148) surveys, with response levels of 81%, 77% and 67%, respectively. The 1995 survey covered individuals aged 16 to 64 years; adults in the 1998 survey were 16 to 74 years and in 2003 there was no age restriction.

Smoking status was established by firstly asking respondents whether they had ever smoked a cigarette, a cigar or a pipe (they were asked "May I just check, have you ever smoked a cigarette, a cigar or a pipe?" and could answer "Yes" or "No"). If they answered "Yes", they were then asked if they currently smoke cigarettes ("Do you smoke cigarettes at all nowadays?" "Yes" or "No") [4] Respondents were classed as current smokers if they answered "Yes" to both these questions. Those who had ever smoked were additionally asked whether they had ever been advised by a medical person to stop smoking altogether because of their health ("(Has a medical person, (*e.g*. doctor or nurse) ever advised you to stop smoking altogether because of your health?" "Yes" or "No"). Information on postcode sector, health board region and socio-economic factors were also available.

### Socio-economic factors

Four socio-economic measures were compatible for all three surveys: the 1991 area based Carstairs index of material deprivation, [23] occupation-based social class [24] of household chief income earner, educational qualifications attained, and economic activity. The Carstairs index is created using four national census data variables from the 1991 Great Britain census, namely: car ownership, household overcrowding, low social class, and male unemployment, at the level of postcode sectors.[23] High Carstairs index values indicate increased levels of area deprivation. Quintiles of the Carstairs index are used for the presentation of distributions; however, the continuous values were used in formal analyses. The possibility of non-linear relationships between area deprivation and smoking was allowed for by the inclusion of a quadratic deprivation term in models. The Registrar General's social class [24] of chief income earner is used to describe the socio-economic status of the household. Social class was considered in three distinct groups: professional/intermediate, skilled (manual/non-manual), partly skilled/unskilled. Respondents' highest educational qualifications were categorised as none, below degree level, or degree/degree level or above. Individuals' economic activity was considered in four groups: employed, unemployed, retired, or economically inactive.

### Statistical methods

Differences in socio-demographic characteristics between Greater Glasgow and the rest of Scotland, and in socio-demographic characteristics by smoking status were assessed by χ^2 ^and χ^2 ^for trend tests. Multilevel logistic regression analyses were performed by sex, unadjusted and adjusted for age and survey year, and socio-economic factors, accounting for the hierarchy of individuals within postcode sector areas. The impact of the differential socio-economic profiles between Greater Glasgow and the rest of Scotland was examined by comparing unadjusted and adjusted odds ratio (OR) estimates. To avoid survey non-response bias, differential weighting combined postcode sector, household and individual probabilities as well as ensuring the weighted sample matched population estimates for age, sex and health boards. Any difference in the relationship between cigarette smoking and Greater Glasgow residence for different survey years was assessed by the joint χ^2 ^testing of the significance of interaction terms in the model. Accounting for individuals advised by a medical person to stop smoking was done in two ways: adjustment was made for anyone advised to stop smoking, and separately, ex-smokers who stopped following advice were excluded from analyses. Since only the age range 16–64 years were common to all three surveys, sensitivity analyses were conducted restricting the sample to these core age groups to check consistency.

Missing smoking status, social class, education, and economic activity data were multiply imputed using the chained equations procedure, [25] with 5 imputations corresponding to 5% of observations with incomplete data.[26] Models were fitted and compared with results from complete case analyses.

Analyses were conducted using MLwiN 2.02 and STATA 9.1 statistical software packages.

## Results

For 1995, 1998 and 2003 surveys combined, 11,075 (44.1%) men and 14,052 (55.9%) women participated. Of all respondents, smoking status was collected for 11,033 (99.6%) men and 14,003 (99.6%) women (Table 1). Among these, 1604 (15%) men and 2186 (16%) women lived in the Greater Glasgow Health Board area. The proportions living in Greater Glasgow were similar in the different age groups. Proportionally, more Glasgow-residing individuals were economically inactive, or had no educational qualifications (*p *=< 0.001); most markedly Greater Glasgow had far larger numbers living in the most deprived Carstairs quintile areas (*p *=< 0.001). However, the social class distribution in Greater Glasgow was no different to that of the rest of Scotland (*p *= 0.204). Compared with the rest of Scotland, Greater Glasgow residence was positively associated with the extent of missingness for social class, economic activity and education.

**Table 1:** Socio-demographics for individuals in Greater Glasgow Health Board region and in the rest of Scotland

		Greater Glasgow	Rest of Scotland	*p*-value	Total
n (%)		n = 3790 (15%)	n = 21,246 (85%)		n = 25,036

Sex	Men	1604 (42)	9429 (44)		11,033 (44)

	Women	2186 (58)	11,817 (56)	0.019	14,003 (56)

Age	16–24	440 (12)	2199 (10)		2639 (11)

	25–34	734 (19)	4048 (19)		4782 (19)

	35–44	819 (22)	4430 (21)		5249 (21)

	45–54	636 (17)	3891 (18)		4527 (18)

	55–64	653 (17)	3820 (18)		4473 (18)

	65–74	387 (10)	2162 (10)		2549 (10)

	75 and over	121 (3)	696 (3)	0.084	817 (3)

Carstairs quintile	Least deprived	709 (19)	4448 (21)		5157 (21)

	2	249 (7)	4739 (22)		4988 (20)

	3	354 (9)	4729 (22)		5083 (20)

	4	485 (13)	4467 (21)		4954 (20)

	Most deprived	1993 (53)	2861 (13)	<0.001	4854 (19)

Social class	I/II	1066 (28)	6330 (30)		7396 (30)

	III	1625 (43)	9484 (45)		11,109 (44)

	IV/V	867 (23)	4733 (22)	0.204	5600 (22)

	Unknown	232 (6)	699 (3)	<0.001^a^	931 (4)

Economic activity status	Employed	1798 (47)	12,175 (57)		13,973 (56)

	Unemployed	221 (6)	924 (4)		1145 (5)

	Retired	582 (15)	3237 (15)		3819 (15)

	Economically inactive	1179 (31)	4887 (23)	<0.001	6066 (24)

	Unknown	10 (0.3)	23 (0.1)	0.015^a^	33 (0.1)

Education	No qualification	1767 (47)	8472 (40)		10,239 (41)

	Below degree level	1388 (37)	9438 (44)		10,826 (43)

	Degree level or above	623 (16)	3308 (16)	<0.001	3931 (16)

	Unknown	12 (0.3)	28 (0.1)	0.009^a^	40 (0.2)

a: *p*-values for *χ*^2 ^test of difference by Greater Glasgow/rest of Scotland residence in proportion of individuals with unknown values

For the whole of Scotland 33% of both men and women were current smokers, whereas prevalence for Greater Glasgow was 38% of men and 40% of women, significantly higher than the 32% for both men and women in the rest of the country (*p *< 0.001) (Table 2). Smoking prevalence peaked in 25–34 year olds then decreased with age, and declined significantly from 1995 to 2003 (*p *< 0.001 for both men and women). Prevalence was significantly associated with deprived area of residence, low social class, unemployment/economic inactivity and low educational attainment (*p *< 0.001 in both men and women for all these characteristics). Although smoking status was not significantly different in men with known and unknown social class (*p *= 0.459), economic activity (*p *= 0.428), or education (*p *= 0.887), nor in women with known and unknown education (*p *= 0.081), it was significantly higher in women with unknown compared with known social class (*p *< 0.001) and economic activity (*p *= 0.006).

**Table 2:** Socio-demographics by smoking status for men and women

Variable		Men			Women		
n (%)		Current Smokers n = 3632 (33%)	Non/Ex-smokers n = 7401 (67%)	*p*-value	Current Smokers n = 4657 (33%)	Non/Ex-smokers n = 9346 (67%)	*p*-value

Area	Greater Glasgow	612 (38)	992 (62)		866 (40)	1320 (60)	

	Rest of Scotland	3,020 (32)	6409 (68)	<0.001	3791 (32)	8026 (68)	<0.001

Age	16–24	438 (37)	753 (63)		550 (38)	898 (62)	

	25–34	802 (39)	1250 (61)		1090 (40)	1640 (60)	

	35–44	812 (34)	1555 (66)		990 (34)	1892 (66)	

	45–54	686 (34)	1327 (66)		865 (34)	1649 (66)	

	55–64	631 (31)	1374 (69)		759 (31)	1709 (69)	

	65–74	215 (20)	865 (80)		345 (23)	1124 (77)	

	75 and over	48 (15)	277 (85)	<0.001	58 (23)	434 (88)	<0.001

Year	1995	1256 (36)	2267 (64)		1665 (38)	2741 (62)	

	1998	1373 (35)	2555 (65)		1763 (35)	3320 (65)	

	2003	1003 (28)	2579 (72)	<0.001	1229 (27)	3285 (73)	<0.001

Carstairs quintile	Least deprived	551 (24)	1773 (76)		635 (22)	2198 (78)	

	2	604 (27)	1611 (73)		778 (28)	1995 (72)	

	3	758 (33)	1519 (67)		915 (33)	1891 (67)	

	4	775 (36)	1372 (64)		1072 (38)	1735 (62)	

	Most deprived	944 (46)	1126 (54)	<0.001	1257 (45)	1527 (55)	<0.001

Social class	I/II	767 (23)	2567 (77)		917 (23)	3145 (77)	

	III	1736 (35)	3223 (65)		2070 (34)	4080 (66)	

	IV/V	1008 (43)	1344 (57)	<0.001	1442 (44)	1806 (56)	<0.001

	Unknown	121 (31)	267 (69)	0.459^a^	228 (42)	1806 (56)	<0.001^a^

Economic activity status	Employed	2068 (30)	4843 (70)		2183 (31)	4879 (69)	

	Unemployed	438 (59)	300 (41)		211 (52)	196 (48)	

	Retired	295 (19)	1249 (81)		513 (23)	1762 (77)	

	Other economically inactive	825 (45)	1001 (55)	<0.001	1738 (41)	2502 (59)	<0.001

	Unknown	6 (43)	8 (57)	0.428^a^	12 (63)	7 (37)	0.006^a^

Education	No qualification	1767 (40)	2641 (60)		2379 (41)	3452 (59)	

	Below degree level	1536 (33)	3185 (67)		1958 (32)	4147 (68)	

	Degree level or above	324 (17)	1564 (83)	<0.001	308 (15)	1735 (85)	<0.001

	Unknown	5 (31)	11 (69)	0.887^a^	12 (50)	12 (50)	0.081^a^

a: *p*-values for *χ*^2 ^test of difference in smoking rates between individuals with complete and those with unknown values

Since the effect of Greater Glasgow residence on current smoking status did not vary significantly by year (*χ*^2^(2 *degrees of freedom (df)*) = 2.167, *p *= 0.338 for men; *χ*^2 ^(2 *df*) = 1.726; *p *= 0.422 for women), it was valid to perform all analyses on combined data from all three survey years. Univariably, compared with the rest of Scotland, men living in Greater Glasgow were 30% more likely to smoke: odds ratio (OR) = 1.30, (95% CI = 1.08–1.56) and women 43% more likely (OR = 1.43, CI = 1.22–1.68) (Table 3). Accounting for age, socio-economic factors and survey year, living in Greater Glasgow was no longer significantly associated with current smoking (OR = 0.92, CI = 0.78–1.09 for men, and OR = 1.08, CI = 0.94–1.23 for women) (Table 3). Among men, results from imputed data analyses were similar: OR = 1.02, CI = 0.88–1.17; however, among women results were borderline significant (OR = 1.15, CI = 1.02–1.29). There was significant variability between postcode sector areas [0.100 (standard error = 0.021) for men and 0.047 (0.015) for women].

**Table 3:** Logistic regression results of current smoking status for Greater Glasgow compared with the rest of Scotland

	Men		Women	
	Odds ratio	95% CI^a^	Odds ratio	95% CI^a^

Unadjusted	1.30	1.08, 1.56	1.43	1.22, 1.68

Age and survey year adjusted	1.30	1.08, 1.57	1.44	1.23, 1.69

Adjusted for age, survey year and socio-economic factors^b^	0.92	0.78, 1.09	1.08	0.94, 1.23

Adjusted for age, survey year, and advised to stop smoking	1.23	1.02, 1.47	1.36	1.15, 1.61

Adjusted for age, survey year socio-economic factors^b^, and advised to stop smoking	0.90	0.75, 1.08	1.08	0.93, 1.25

^a^: 95% Confidence interval

^b ^Carstairs quintile, Social class, Economic activity status, Education

The possible effect of individuals changing their habit on medical advice was assessed by both adjusting for advice to stop smoking and by excluding ex-smokers who stopped following medical advice. Adjusting for advice to stop smoking resulted in only minor changes to the estimates (Table 3); again similar results were found among men (OR = 0.99, CI = 0.86–1.16) but borderline significant in women (OR = 1.15, CI = 1.02–1.31) from analyses of imputed data. Similarly, excluding ex-smokers who stopped following medical advice (555 men and 514 women) did not substantially alter results (Table 4); once more, similar results were found in men (OR = 1.02, CI = 0.88–1.17) but borderline significant among women (OR = 1.15, CI = 1.02–1.29) in analyses of imputed data. Finally, restricting analyses to the core 16–64 year age groups yielded equivalent results.

**Table 4:** Current smoking status logistic regression results, excluding those who stopped following medical advice

	Men		Women	
	Odds ratio	95% CI^a^	Odds ratio	95% CI^a^

Unadjusted	1.30	1.08, 1.57	1.44	1.22, 1.69

Age and survey year adjusted	1.31	1.08, 1.58	1.44	1.23, 1.70

Age and socio-economic factors^b ^adjusted	0.92	0.77, 1.09	1.08	0.94, 1.23

^a^: 95% Confidence interval

^b ^Carstairs quintile, Social class, Economic activity status, Education

## Discussion

Although smoking prevalence in Greater Glasgow is elevated compared with the rest of the country, we have shown this to be due to adverse socio-economic circumstances in the city. That Greater Glasgow has an unfavourable health-related behaviour profile compared with other parts of Scotland is not surprising. This also holds for urban comparisons – in relation to Edinburgh, prevalences of smoking and other cardiovascular disease risks are elevated in Glasgow [27]. What was previously uncertain was the extent to which this health disadvantage was due to the particularly deprived socio-economic composition of the area.

A previous study comparing regions in Scotland found not Glasgow, but the relatively affluent Perth and Kinross region, at the top of the league for cardiovascular risk factors – smoking being one of the major contributing factors.[28]. However, this analysis was based on the working population, excluding economically inactive individuals – the group most likely to smoke. Since Glasgow has 30% (compared with 21% nationally) of those of working age economically inactive [29] the Glasgow sample in that study was more like samples from other regions than would have been the case if the study was based on the general population, and is likely to have underestimated smoking rates in Glasgow. This concurs with our findings that it is Greater Glasgow's higher numbers of deprived areas and economic inactivity that elevate smoking rates in the area relative to other parts of the country, and that smoking rates are not higher in Greater Glasgow when comparing economically similar groups.

There were a number of limitations to the survey data used for this study which should be acknowledged. Smoking status in the Scottish Health Survey is ascertained by self-report which is known to underestimate true prevalence [30]. Although figures obtained may not be a reliable indicator of actual rates, they should be comparable across region groups within the same survey, which was confirmed by investigation of levels of cotinine, the nicotine metabolite present in saliva. Smoking-cotinine associations were found to be equivalent in Greater Glasgow and the rest of Scotland (*p *= 0.755, data not shown). It may be that among smokers, cigarette consumption is higher in Glasgow than the rest of the country. It is known, for instance, that nicotine intake among smokers is higher in lower socio-economic groups, [31] and that smokers in Scotland have higher nicotine intakes than those in England, even within socio-economic deprivation groups; [32] this may be paralleled in concentration within Scotland, in the Glasgow area. Measures of socio-economic status used were cross-sectional, which may not adequately capture the effects of socio-economic circumstances on morbidity and mortality.[33] However, smoking has been shown to be associated primarily with current as opposed to past socio-economic circumstances [34] and so the measures used here are likely to be appropriate. The data were collected during three separate surveys spanning eight years, over which time smoking prevalence decreased, as did survey response levels. However, as indicated by the non-significance of the Greater Glasgow residence by year interaction, the relationship between smoking and Greater Glasgow residence did not significantly differ across surveys, validating the combination of data from all surveys.

Strengths of this study include breadth of socio-economic indicators, representativeness and the large sample size. Since a broad range of measures – covering area deprivation, household social class, individual economic activity and education attainment – was used, a comprehensive socio-economic profile has been captured, minimizing problems with measurement inadequacies. Analyses are based on rigorously collected data on over 25,000 individuals, characteristic of the general population, for whom smoking status was reported in all but a small number of cases. Finally, the additional analyses conducted accounting for medical advice to stop smoking and excluding ex-smokers provide robust evidence to support the findings that socio-economic circumstances explain elevated smoking levels in the Glasgow area compared with the rest of Scotland. There were higher levels of missing social class, economic activity and education data in Greater Glasgow, and smoking status was significantly higher in women with unknown social class and economic activity, underlining the need for sensitivity analyses based on multiply imputed data. Contrary to the tabulated complete case analyses results, those based on imputed data remained borderline significant for women, indicating some bias in the incomplete cases. However, overall, analyses based on imputed data confirmed attenuation in both men and women.

## Conclusion

In summary, individual and area socio-economic circumstances drive elevated smoking levels in the Glasgow area compared with the rest of Scotland, especially among men, reflecting its poorer socio-economic position. Within socially homogenous groups, smoking rates were similar in Greater Glasgow compared with the rest of the country.

Since the strong social patterning of cigarette smoking in Greater Glasgow is equivalent to that seen elsewhere in Scotland, addressing the poor health of the population – at least in terms of smoking-related diseases – extends beyond Glasgow to national requirements to address the high levels of smoking (and the causes of smoking) among the most disadvantaged social groups. Thus tackling smoking-related health problems requires policies to address poverty, not just in Greater Glasgow but in Scotland as a whole. Future research could investigate to what extent higher smoking rates and other health risk behaviours explain high morbidity and mortality in the Glasgow area, and the degree to which associations are mediated by deprivation.

## Competing interests

The authors declare that they have no competing interests.

## Authors' contributions

Formulation of the research question arose from discussion between both authors. Analyses were planned and performed, and the draft of the manuscript was prepared by LG. AL contributed to the writing of the manuscript. All authors read and approved the final manuscript.

## Pre-publication history

The pre-publication history for this paper can be accessed here:


